# Can microprocessor knees reduce the disparity in trips and falls risks between above and below knee prosthesis users?

**DOI:** 10.1371/journal.pone.0271315

**Published:** 2022-09-02

**Authors:** Michael McGrath, Laura A. Gray, Beata Rek, Kate C. Davies, Zoe Savage, Jane McLean, Alison Stenson, Saeed Zahedi

**Affiliations:** 1 Blatchford, Basingstoke, United Kingdom; 2 School of Health and Related Research, University of Sheffield, Sheffield, South Yorkshire, United Kingdom; 3 Sheffield Teaching Hospitals, Sheffield, South Yorkshire, United Kingdom; West Park Healthcare Centre, CANADA

## Abstract

While all lower limb prosthesis walkers have a high risk of tripping and/or falling, above knee prosthesis users are reported to fall more frequently. Recognising this, engineers designed microprocessor knees (MPK) to help mitigate these risks, but to what extent these devices reduce this disparity between above and below knee users is unclear. A service review was carried out in a prosthetic limb centre regarding the frequency of trips and falls in the previous four weeks. Data from unilateral, community ambulators were extracted. Ordered logistic regressions were applied to investigate whether MPKs mitigated the increased risk of trips and falls for prosthetic knee users, compared to below knee prosthesis users. Socio-demographics (sex, age), prosthesis (prosthesis type, years of use), health (comorbidities, vision, contralateral limb status, medication), and physical function (use of additional walking aids, activity level) were included as covariates. Of the 315 participants in the analysis, 57.5% reported tripping and 20.3% reported falling. Non-microprocessor prosthetic knee (non-MPK) users were shown to trip significantly more than below knee prosthesis users (OR = 1.96, 95% CI = 1.17–3.28). Other covariates showing a significant association included contralateral limb injuries (OR = 1.91, 95% CI = 1.15–3.18) and using an additional walking aid (OR = 1.99, 95% CI = 1.13–3.50). Non-MPK users were also shown to fall significantly more than below knee prosthesis users (OR = 3.34, 95% CI = 1.73–6.45), with no other covariates showing a significant association. MPK users did not show an increased frequency of trips (OR = 0.74, 95% CI = 0.33–1.64) or falls (OR = 0.34, 95% CI = 0.18–2.62), compared to below knee prosthesis users. Of those who tripped at least once in the previous four weeks, those using a non-MPK (OR = 2.73, 95% CI = 1.30–5.74) presented an increased frequency of falling. These findings provide evidence to suggest that the use of MPKs reduces the difference in falls risk between above knee and below knee prosthesis users, providing justification for their provision.

## Background

People who walk with a lower limb prosthesis in the community face a high probability of falling [1–7]. The loss of joint control and reduced limb proprioception create difficulties with maintaining stability and avoiding tripping hazards. There are often serious consequences for the faller, including soft tissue injuries [1], bone fractures and traumatic head injuries [8,9]. The direct medical costs in the six months after an above knee prosthesis user falling have been estimated as being as high as $39,000 (USD), potentially rising as high as $53,000 (USD) if an emergency department admission is required [10].

Other research has also indicated that this risk is heightened for those with more proximal limb absence [1–3]. While the rate of annual falls is up to 53% for below knee prosthesis users, it can be up to 66% for above knee prosthesis users [1,2]. A study by Miller et al. in the early 21^st^ century, determined that above knee prosthesis users had nearly three times the odds of falling in a year, compared to below knee prosthesis users [2].

Since then, however, there have been many advancements in prosthetic knee technology, with design engineers focussing on producing devices that enhance stability and mitigate the risk of falls. Microprocessor knees (MPKs) are reported to reduce the incidence of falls for above knee prosthesis users. MPKs perform adaptations to the resistance to knee flexion during each gait cycle, providing greater stance phase stability and bodyweight support. Further recent innovations include functionality to detect aberrant steps and adjust the knee resistance accordingly, enabling sudden prosthetic weight-bearing to recover from a stumble. These changes may explain users of MPKs reporting a reduced frequency of stumbles [11] and a lower frequency of falls [12]. These findings contributed to the justification of the provision of these devices by the National Health Service in England (NHSE) starting in 2016 [13]. Since then, a larger study of 602 MPK users from the US has reiterated the finding of a significant reduction in injurious falls [14]. Chen et al.’s modelling study reported that for 100 people over 10 years, MPKs have been shown to reduce direct healthcare costs per person by $3676 and even save 11 lives, largely due to the reduction in falls risk [15].

It is not clear, however, the extent to which this improvement mitigates the disparity in trips and falls risk between above knee and below knee prosthesis users. The objective of this work was to examine the frequency of trips and falls of unilateral, community-ambulating, lower limb prosthesis users and investigate the associations with prosthesis type. Moreover, it was hypothesised that the use of MPKs would reduce the difference in falls rate between above knee and below knee prosthesis users.

## Methods

### Data sample

The dataset for this analysis was extracted from a ‘Trips, Stumbles and Falls Questionnaire’ (S1 Appendix), during a service review of all lower limb prosthesis users at a prosthetic limb centre in Sheffield, UK, collected over a three month period. The main outcomes were how often respondents had tripped/stumbled and how often they had fallen within the prior four weeks. A trip was defined as *‘catching one’s foot on something*, *leading to a stumble or fall’* while a stumble was defined as *‘momentarily losing one’s balance; almost fall’*. The number of trips and stumbles was collected as a single variable. A fall was defined as *‘losing one’s balance and collapsing to the ground’*. The 4-week time period was chosen to be equivalent to that used by the Prosthesis Evaluation Questionnaire [16] (PEQ) and the PEQ-Addendum [6,7] (which specifically relates to balance, trips and falls). While other research has reported self-reported falls over the prior 12 month period [1,2], it was felt that a timeframe beyond four weeks could be affected by recall errors [7]. Responses were given in ordinal categories of *‘None’*, *‘1–5 times’* or *‘More than 5 times’*.

The Clinical Effectiveness Unit of Sheffield Teaching Hospitals NHS Foundation Trust provided ethical approval (project reference 7537; S2 Appendix). Participants provided verbal consent and completed anonymous paper questionnaires during their routine clinical appointments. The data were analysed anonymously.

The focus of this analysis was on unilateral, prosthetic community ambulators. From the original dataset, to ensure the participants could be described as “community ambulators”, only those with an activity level of K2 or higher were included, as determined by their prosthetist. A K2 ambulator is described as *‘having the ability or potential for ambulation with the ability to traverse low-level environmental barriers such as curbs*, *stairs or uneven surfaces’* [17]. Furthermore, any bilateral users were excluded. Finally, to ensure that all participants used a prosthetic foot or a prosthetic foot and knee, those categorised as having *‘Partial foot/ankle disarticulation’* limb absences were also excluded.

### Prosthetic devices

Each participant in the cohort was assigned a *‘Prosthesis type’*, which was divided into those who used a below knee prosthesis and those who used a prosthetic knee (inclusive of those with knee disarticulation, transfemoral and hip disarticulation/hemipelvectomy limb absences). While data regarding the types and technologies of the prosthetic components were not explicitly collected during the service review, some of the prosthetic knee user participants were known to be partaking in the NHS England Microprocessor Knee Commissioning Policy [13]. As a prerequisite of this commissioning policy, patient outcome measures must be collected. For these participants, the responses to this ‘Trips, Stumbles and Falls’ questionnaire were retained in their outcome measure records. For this reason, the prosthetic device type of these participants could be identified as an MPK (Orion3, Blatchford, Basingstoke). Consequently, the prosthetic knee users were divided into two further categories; *‘non-MPK’* and *‘MPK’*.

### Key outcomes

The outcomes of interest in this analysis were the frequency of trips/stumbles (collected as a single variable) and falls within the previous four-week period. Participants selected responses of *‘None’*, *‘1–5 times’* and *‘More than 5 times’*.

### Covariates

In order to provide evidence that any observations were not being adversely influenced by confounding factors, other covariates were considered in each of these analyses, reflecting a number of characteristics that may affect falls risk.

Sociodemographic covariates included sex and age, both of which have been previously associated with falls risk [18,19]. Due to relatively low numbers of participants aged under 20 years (n = 4), this category was merged with the adjacent category for the analysis. In effect, this left four age brackets; *‘18–39 years’*, *‘40–59 years’*, *‘60–79 years’* and *‘>80 years’*.

With regard to the participants’ experience using a prosthesis, *‘Years of prosthesis use’* was included as another covariate. The length of time that someone has been a prosthesis user has been previously reported to affect the frequency of falls [2]. The categories included *‘<1 year’*, *‘1–5 years’*, *‘6–10 years’* and *‘>10 years’*.

Health-related covariates previously linked to the risk of falls included comorbidities known to affect balance [19] and the use of medication with loss of balance or dizziness as a potential side effect [19]. While data were collected in relation to specific comorbidities (see S1 Appendix), this analysis dichotomised these data into the presence or absence of at least one comorbidity, owing to the fact that there were insufficient numbers of participants with each individual comorbidity. Similarly, whether the participant had an injury on their contralateral (not affected by limb absence) limb was included as a covariate. While vision is also known to influence falls risk and these data were collected [20], among the unilateral, community ambulators, there was a limited number with vision issues (n = 8) so this was removed as a covariate.

Finally, covariates related to physical function included the use of walking aids (additional to the prosthetic limb) and prosthetist-assessed K level of mobility. Data regarding the use of specific walking aids were refined into a binary *‘Yes’* or *‘No’*, while the K level categories were *‘K2’*, *‘K3’* (*‘a community ambulator who also has the ability to traverse most environmental barriers … ability or potential for ambulation with variable cadence’*) and *‘K4’* (*‘ability or potential for prosthetic ambulation that exceeds basic ambulation skills*, *exhibiting high impact*, *stress or energy levels’*) [17].

### Data analyses

Three ordered logistic regression analyses were performed to analyse the data. The first estimated the frequency of trips/stumbles, the second analysis estimated the frequency of falls and the final analysis included only those who reported at least one trip in the previous four weeks, again using the frequency of falls as the dependent variable. Only those participants that had complete data for all of the covariates were extracted from the original dataset for this analysis. Ordered logistic regressions were performed using the statistical software *R* v4.0.0 (The R Foundation, Vienna, Austria) and odds ratios and 95% confidence intervals are provided.

## Results

**Fig 1:**
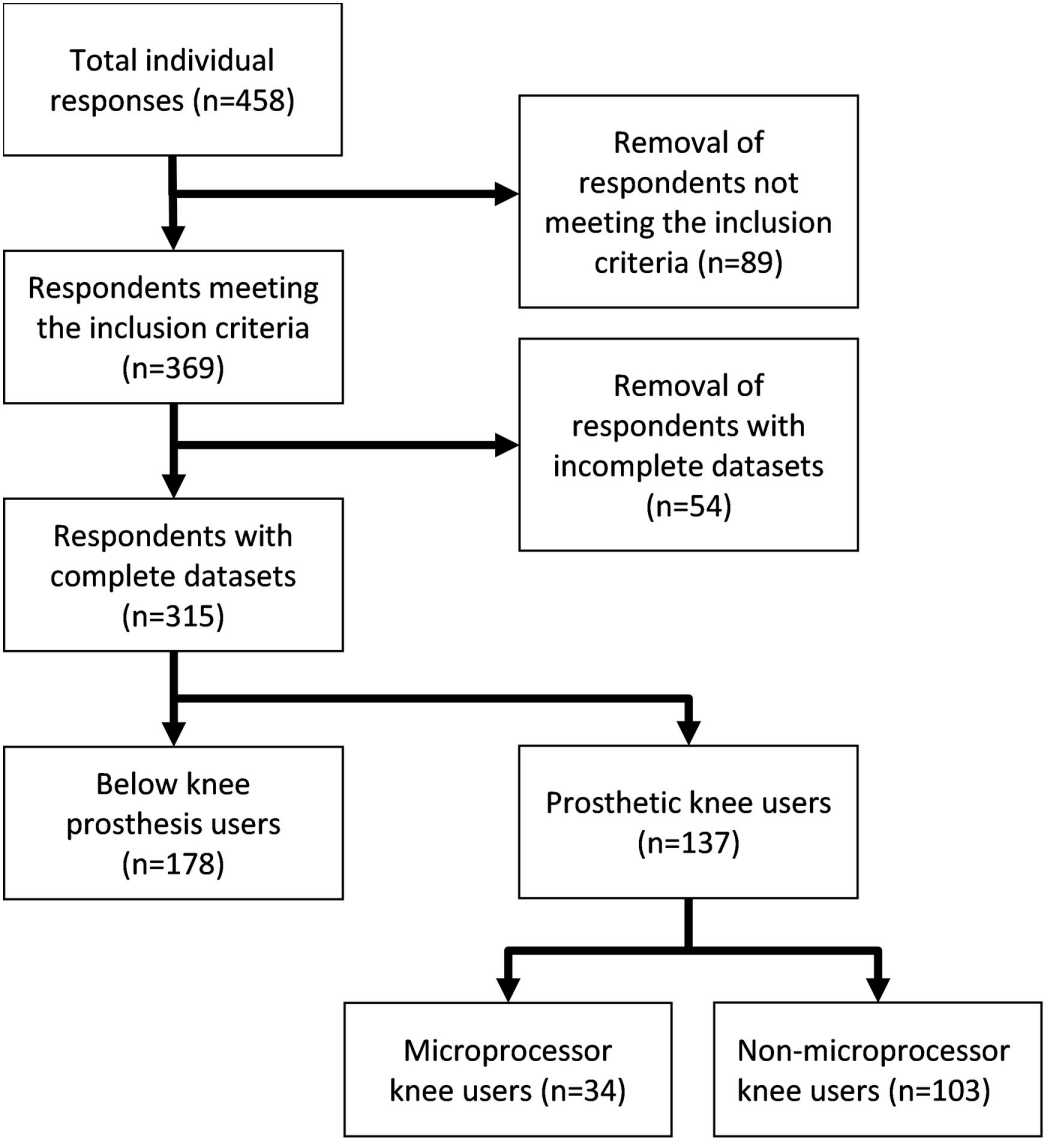
Flowchart showing the breakdown of the study cohort.

There were 458 responses to the questionnaire. After excluding 89 who did not meet the study inclusion criteria and a further 54 with missing data, a total of 315 participants were included in the analyses (Fig 1). The cohort was 80.1% male with 82.0% in either the *‘40–59 years’* or *‘60–79 years’* age brackets. In terms of their prosthesis, 56.5% wore a below knee prosthesis. Of the prosthetic knee users, 24.8% had MPKs. The majority (66.0%) had been using a prosthesis for over 10 years. Comorbidities affecting balance were reported by 21.0%, medication affecting balance was reported by 9.5% and a contralateral limb injury was reported by 27.3%. Approximately half the participants (50.2%) said they sometimes used a walking aid, additional to their prosthesis. K2 (47.9%) and K3 (42.2) activity levels were the most common, with K4 users composing the remainder of the study group.

At least one trip in the previous four weeks was reported by 57.5%, while 20.3% reported one or more falls in the same period.

### Tripping analysis

The frequencies of trips/stumbles for users of different prosthesis types, and each of the covariate groups, are shown in Table 1. The odds ratios and 95% confidence intervals resulting from the ordered logistic regression are included.

**Table 1:** The frequency of trips/stumbles by prosthesis type and each of the covariates. Odds ratios (OR) and 95% Confidence Intervals (CI) are shown; bold text indicates statistically significant observations.

	*None (n = 134)*	*1–5 times (n = 145)*	*More than 5 times (n = 36)*	*OR*	*95% CI*
***Prosthesis type***					
Below knee prosthesis users	84	78	16	1.00	
Non-microprocessor knee users	32	53	18	**1.96**	**1.17–3.28**
Microprocessor knee users	18	14	2	0.74	0.33–1.64
***Covariates***					
**Sociodemographic**					
*Sex*					
Male	108	118	27	1.00	
Female	26	27	9	1.03	0.58–1.83
*Age*					
18–39 years	21	23	3	1.00	
40–59 years	48	66	15	0.99	0.50–1.96
60–79 years	61	50	18	0.80	0.40–1.64
>80 years	4	6	0	0.65	0.17–2.48
**Experience**					
*Years of prosthesis use*					
>10 years	94	90	24	1.00	
6–10 years	16	19	3	1.01	0.50–2.05
1–5 years	14	32	8	1.62	0.85–3.07
<1 year	10	4	1	0.43	0.13–1.40
**Health**					
*Comorbidity (-ies)*					
No	106	115	28	1.00	
Yes	28	30	8	0.89	0.50–1.57
*Contralateral limb injury*					
No	107	101	21	1.00	
Yes	27	44	15	**1.91**	**1.15–3.18**
*Medication*					
No	125	128	32	1.00	
Yes	9	17	4	1.16	0.55–2.45
**Physical function**					
*Walking aid(s)*					
No	79	66	12	1.00	
Yes	55	79	24	**1.99**	**1.13–3.50**
*Activity level*					
K2	58	75	18	1.00	
K3	63	56	14	1.28	0.72–2.29
K4	13	14	4	1.53	0.64–3.68

Non-MPK users were more likely to trip than below knee prosthesis users (OR = 1.96, 95% CI = 1.17–3.28). However, there was no significant difference in the likelihood of tripping between below knee prosthesis users and MPK users (OR = 0.74, 95% CI = 0.33–1.64).

In terms of the other covariates, those with an injury on the contralateral limb were more likely to trip than those without (OR = 1.91, 95% CI = 1.15–3.18) and those who used an additional walking aid also had a greater probability of tripping than those who walked only with a prosthesis (OR = 1.99, 95% CI = 1.13–3.50).

### Falling analysis

The frequencies of falls for users of the different prosthesis types, and for each of the covariate groups, are shown in Table 2, along with results from the ordered logistic regression.

**Table 2:** The frequency of falls by prosthesis type and each of the covariates. Odds ratios (OR) and 95% Confidence Intervals (CI) are shown; bold text indicates statistically significant observations.

	*None (n = 251)*	*1–5 times (n = 57)*	*More than 5 times (n = 7)*	*OR*	*95% CI*
***Prosthesis type***					
Below knee prosthesis users	154	22	2	1.00	
Non-microprocessor knee users	66	32	5	**3.34**	**1.73–6.45**
Microprocessor knee users	31	3	0	0.68	0.18–2.62
***Covariates***					
**Sociodemographic**					
*Sex*					
Male	201	45	7	1.00	
Female	50	12	0	0.78	0.36–1.68
*Age*					
18–39 years	42	5	0	1.00	
40–59 years	95	31	3	1.74	0.59–5.07
60–79 years	106	19	4	1.07	0.35–3.32
>80 years	8	2	0	1.06	0.15–7.36
**Experience**					
*Years of prosthesis use*					
>10 years	166	38	4	1.00	
6–10 years	31	5	2	1.16	0.44–3.10
1–5 years	41	12	1	1.09	0.47–2.50
<1 year	13	2	0	0.76	0.14–4.11
**Health**					
*Comorbidity (-ies)*					
No	200	42	7	1.00	
Yes	51	15	0	1.13	0.55–2.32
*Contralateral limb injury*					
No	186	40	3	1.00	
Yes	65	17	4	1.47	0.77–2.83
*Medication*					
No	232	47	6	1.00	
Yes	19	10	1	1.72	0.71–4.15
**Physical function**					
*Walking aid(s)*					
No	133	22	2	1.00	
Yes	118	35	5	1.51	0.71–3.21
*Activity level*					
K2	115	33	3	1.00	
K3	111	18	4	0.94	0.44–2.01
K4	25	6	0	1.12	0.35–3.54

Non-MPK users have over three times greater odds of falling than below knee prosthesis users (OR = 3.34, 95% CI = 1.73–6.45), but there was no significant difference in the fall frequency of MPK users and below knee prosthesis users (OR = 0.68, 95% CI = 0.18–2.62). None of the other covariates indicated a significant association.

### Falling analysis of those who reported a trip

When considering only those that reported at least one trip in the previous four weeks, the frequencies of falls for users of each prosthesis type, and for each of the covariate groups, are shown in Table 3. The odds ratios and 95% confidence intervals resulting from the ordered logistic regression are included.

**Table 3:** The frequency of falls among those that reported at least one trip/stumble in the previous 4 weeks, ordered by prosthesis type and each of the covariates. Odds ratios (OR) and 95% Confidence Intervals (CI) are shown; bold text indicates statistically significant observations.

	*None (n = 120)*	*1–5 times (n = 54)*	*More than 5 times (n = 7)*	*OR*	*95% CI*
***Prosthesis type***					
Below knee prosthesis users	71	21	2	1.00	
Non-microprocessor knee users	35	31	5	**2.73**	**1.30–5.74**
Microprocessor knee users	14	2	0	0.34	0.06–1.85
***Covariates***					
**Sociodemographic**					
*Sex*					
Male	94	44	7	1.00	
Female	26	10	0	0.57	0.24–1.38
*Age*					
18–39 years	21	5	0	1.00	
40–59 years	49	29	3	1.59	0.50–5.09
60–79 years	46	18	4	1.24	0.37–4.13
>80 years	4	2	0	0.71	0.09–5.71
**Experience**					
*Years of prosthesis use*					
>10 years	73	37	4	1.00	
6–10 years	15	5	2	1.03	0.35–3.05
1–5 years	29	10	1	0.64	0.25–1.63
<1 year	3	2	0	0.84	0.11–6.62
**Health**					
*Comorbidity (-ies)*					
No	96	40	7	1.00	
Yes	24	14	0	1.03	0.45–2.37
*Contralateral limb injury*					
No	79	40	3	1.00	
Yes	41	14	4	0.93	0.44–1.97
*Medication*					
No	109	45	6	1.00	
Yes	11	9	1	1.21	0.43–3.39
**Physical function**					
*Walking aid(s)*					
No	56	20	2	1.00	
Yes	64	34	5	1.64	0.70–3.86
*Activity level*					
K2	59	31	3	1.00	
K3	49	17	4	1.15	0.50–2.64
K4	12	6	0	1.06	0.29–3.90

When non-MPK users reported a trip, they were significantly more likely to also report a fall than below knee prosthesis users that had tripped (OR = 2.73, 95% CI = 1.30–5.74). When MPK users reported a trip, they were no more likely than below knee *‘trippers’* to fall (OR = 0.34, 95% CI = 0.06–1.85). None of the other covariates had a statistically significant association.

## Discussion

This analysis provided insights into how prosthetic technology is associated with the incidence of trips and falls in unilateral, community-ambulating prosthesis users. Furthermore, the findings provided evidence to support the study hypothesis, that the use of MPKs can help to mitigate the increased risk of trips and falls previously reported for above knee prosthesis users, compared to below knee prosthesis users [1,2].

Of the non-MPK users, 68.9% reported at least one trip and 35.9% reported at least one fall in the prior four weeks, compared to 52.8% and 13.5%, respectively, for the below knee prosthesis users. These trips and falls rates for MPK users were 47.1% and 8.8%, respectively; more comparable to those of below knee prosthesis users than those with the same level of limb absence without microprocessor technology. Indeed, there were no significant differences between MPK users and below knee prosthesis users.

A greater falls prevalence has often been reported for those with more proximal levels of limb absence [1,2]. It is notable, however, that much of this research was performed prior to the wide-spread provision of stance-and-swing control MPKs, which were not commercially available until the late 1990s. Both crossover studies [12] and cross-sectional studies [14] of MPKs and non-MPKs have reported significant reductions in the incidence of falls with MPKs. To the authors’ knowledge, none have investigated how this improvement in falls rate compares to the outcomes of below knee prosthesis users. In terms of mobility, the use of MPKs has been shown to mitigate some of the disparity between below knee and above knee prosthesis users [21], which may have an influence on the likelihood of tripping and falling.

While the exact, direct cause of each reported fall was not recorded, the final analysis evaluated only those who had reported at least one trip, to see if there were any characteristics of people who tripped that were protective against falling (Table 3). Non-MPK users who tripped were significantly more likely to fall than below knee prosthesis users who tripped (no other covariates had a significant association). One explanation for this might be that when the trip occurs on the prosthetic foot and the user tries to quickly recover and bear weight on that limb, the knee joint is likely to be flexed with the ground reaction force vector acting posterior to the knee joint centre. The below knee prosthesis user retains muscular function to counteract the resulting external flexion moment, providing support and preventing the limb from buckling. In contrast, the non-MPK user cannot generate a moment to resist knee flexion and so the limb does not provide adequate support. Modern MPKs provide a ‘stumble recovery’ functionality, whereby the device provides a high knee flexion resistance, should a perturbation have a sudden impact on swing phase mechanics. This allows the user to bear weight on the flexed prosthetic limb, even when the ground reaction vector acts posterior to the knee joint centre. In this analysis, there was no significant difference in the likelihood of falling between below knee prosthesis *‘trippers’* and MPK *‘trippers’*.

These analyses also accounted for a number of covariates. The tripping analysis showed an association between the use of a walking aid, additional to their prosthetic limb, and the probability of tripping (Table 1). It might be speculated that those using walking aids may be doing so due to a self-awareness of their propensity to trip. A notable observation, however, is it 41.2% of the MPK users reported using an additional walking aid, despite their advanced prosthetic technology. Perhaps a limitation of this analysis is the lack of information available regarding how long the MPK users had been using that specific prosthesis. The NHS England MPK Clinical Commissioning Policy [13], under which these devices had been supplied, stipulates a minimum of four weeks acclimatisation before outcome measures are gathered, equivalent to that of the timeframe of the collected data in this analysis. However, data are not available to determine whether each MPK user had been using their device for only four weeks or if they had used it for months. The high rate of using additional walking aids may be explained by users who have always used walking aids with their previous, non-MPK and have not broken the habit. Perhaps these users are also the ones who have less experience with their MPK, which could be a factor in the significantly higher rate of tripping. However, without data available, this can only be speculated. Furthermore, since this was a cross-sectional analysis, the data do not indicate any progressive change in the requirements for additional walking aids. For example, an MPK user that uses a crutch as a walking aid, may have previously required two crutches or a walking frame when they didn’t have the advanced prosthesis.

An injury or condition affecting the contralateral limb was also identified as a risk factor for tripping (Table 1). Such injuries may lead trips caused by catching the sound foot, due to inadequate swing phase ground clearance on the contralateral limb. They might also increase the likelihood of tripping with the prosthetic foot, as it is more difficult to perform compensatory gait movements, such as vaulting, which are commonly employed by lower limb prosthesis users to ensure clearance of the prosthetic limb. Reduced sound limb loading during walking has been reported for advanced prosthetic technology, such as MPKs [22] and hydraulic ankles [23], which may serve to reduce the impact of contralateral injuries.

It has been reported that increasing age is a common risk factor for falls, shared by both lower limb prosthesis users and the general population of older adults [19]. However, in the current research, although there was no statistical difference in falls risk between the age groups, the *‘40–59 years’* category had a higher odds ratio than the other groups (Table 2). Perhaps notably, this age group represented the largest proportion– 48.5%–of the non-MPK users. Comparatively, only 37.67% of below knee and 35.3% of MPK users were in that same category. The biggest difference in age distribution between *‘Prosthesis type’* was in the ‘*18–39 years’* category. Only 3.9% of non-MPK users fell into this age band, compared to 19.7% and 23.5% of below knee and MPK users, respectively. Further research might consider investigating disparities in prosthetic prescription practices across different age ranges and the influence this may have on user outcomes.

In terms of tripping, there was a weak relationship to suggest younger, higher mobility users have a higher risk of trips (Table 1), albeit not statistically significant. While the risk factors for tripping in prosthesis users are unclear, it might be hypothesised that the more activity that a person does, the higher the likelihood that they will experience a trip. Additionally, higher mobility may lead to greater confidence and therefore increased risk taking. It is recommended that this area be further explored.

### Limitations

This study used a shorter time frame (4 weeks rather than 12 months) than previous studies [1–5] in order to try to maximise recall. This is the likely reason for the prevalence of falls being lower than the rates of over 50% reported previously [1–5]. One study using 4-week timeframe has reported a 50% falls rate [4], while others only reported mean [6] or median [7] number of falls across the cohort, preventing comparison. Research into falls during inpatient rehabilitation following lower limb amputation [24] reported a closer falls rate (20.5%) to the current study. Although the timeframes in that research were variable (the length of each patient’s hospital stay), they were closer to 4 weeks than 12 months. Indeed, stays of 22–35 days and those greater than 35 days were associated with greater falls risk [24]. However, the specific demographic in that study (inpatient, primary amputees) limits comparability with the current work. Further investigation is warranted to determine how the study timeframe influences any results.

An alternative approach that has been used previously to aid participant recall is collect the number of *injurious* falls; those for which medical attention was required [14]. Intuitively, the most important incentive for reducing the risk of falls among lower limb prosthesis is to lower the prevalence of falls-related injuries. Indeed, one cost-effectiveness analysis of MPKs, compared to non-MPKs, reported for every 100 users, over a ten year time horizon, there would be 82 fewer major injurious falls, 62 fewer minor injurious falls and hence 11 fewer deaths [15]. This was used as justification for their wider provision [15]. A focus on injurious falls helps to create more objective and accurate data but would require analysis of documented medical records. However, when using a self-report questionnaire method like in the current study, although falls resulting in an injury might be more memorable for most, recall inaccuracies cannot be eliminated completely. Furthermore, while the collection of only injurious events may be advisable for the ‘Number of falls’ variable, it would not be possible for the ‘Number of trips/stumbles’ variable.

The questionnaire used pre-determined categories for some continuous data. The rationale behind this was to help preserve participant anonymity (e.g. age) or again to help recall (e.g. number of falls). However, it does have the effect of loss of information, with regard to certain variables. The collection of the dependent variables (numbers of trips or falls) as pre-defined categories is arguably the most limiting factor. By using categories for these, along with an ordered logit model, it must be assumed that the effect of a covariate on the probability of moving from 0 to 1 (i.e., *‘None’* to *‘1–5 times’*) is the same as moving from 1 to 2 (i.e., *‘1–5 times’* to *‘More than 5 times’*). In reality, this may not be the case.

In retrospect, it may also have been prudent to include amputation aetiology as a covariate. Prosthesis users who have undergone dysvascular amputations have been associated with a greater probability of falling than those with a traumatic aetiology [19], but the use of MPKs by this specific user group have reported benefits [25,26]. Reduced fear of falling, increased balance confidence, improved performance in clinical tests related to balance ability [25] and a decreased rate of falls have all been observed when dysvascular prosthetic knee users transitioned to MPKs [25,26]. Future data gathering might consider including aetiology of limb absence to enable such analyses of the dataset.

Further to this, although data regarding comorbidities that may affect balance were collected separately, due to the low participant numbers in each category, the final analysis grouped responses into *‘No comorbidities’* and *‘At least one comorbidity’*. This, unfortunately, does not account for the impact that different comorbidities have on balance and the risks of falling.

Future investigations into the falls risks of prosthesis users might also be advised to consider the circumstances surrounding falls and the effects of the environment. The current study limited its analysis to only those with sufficient mobility capabilities to be described as ‘community ambulators’, but beyond that, information on participants’ degrees of exposure to external influences on falls risk is limited.

Another potential covariate, for which data were not available, was the type of prosthetic foot. It was known that all of the MPK users were fitted with hydraulic ankles (Echelon, Blatchford, Hampshire, UK), in addition to their MPK, as part of a deal provided by the MPK manufacturer. Anecdotally, the prosthetic team in this limb centre reported that it is unlikely for a non-MPK user to be fitted with a hydraulic ankle, but it cannot be guaranteed for all of the non-MPK users in the dataset. Meanwhile, below knee prosthesis users are likely to be fitted with a hydraulic ankle but, once more, because the data were not recorded, the proportion of users in this dataset is unknown. There is published evidence that hydraulic ankles present increased ground clearance during swing [27,28] and enhanced standing stability [29], compared to feet that are rigidly-attached at the ankle. More recently, some researchers have investigated how the combinations of advanced prosthetic knees and ankles work together, suggesting the combination of the two technologies may have biomechanical advantages for balance [29], mobility [30] and navigating everyday, environmental barriers, such as stairs and slopes [30]. It is possible that these characteristics may have provided the MPK users with further protection from trips and falls, compared to non-MPK users that did not have hydraulic ankles, but it is not possible to draw definitive conclusions from the current analyses.

## Conclusion

The findings of this analysis concur with previously published evidence suggesting that above knee prosthesis users have a significantly higher likelihood of tripping and falling than below knee prosthesis users. However, the results also suggested that the use of advanced MPKs could mitigate these heightened risks. This work contributes further to the growing body of evidence to support the prescription of MPKs to enhance stability and reduce falls-related injuries among lower limb prosthesis users.

## Supporting information

S1 Appendix Trips, stumbles and falls questionnaire. (PDF)

S2 Appendix Ethical approval. (PDF)

S3 Appendix Ordered logistic regression full outputs. (PDF)
