# Supplementary material for: Can microprocessor knees reduce the disparity in trips and falls risks between above and below knee prosthesis users?
Source: PLoS One. 2022 Sep 2;17(9):e0271315. doi: 10.1371/journal.pone.0271315 (PMC9439191; doi:10.1371/journal.pone.0271315)
Supplement: S1 Appendix — (PDF) [file pone.0271315.s001.pdf]

Trips, Stumbles and Falls Questionnaire

We are interested in learning about how often amputees trip, stumble or fall in day-to-day life. We would be very grateful if you could take the time to complete the following short questionnaire. Please tick the boxes as appropriate.

1. Are you?

☐ Male ☐ Female

2. How old are you?

☐ 0 - 19 years ☐ 20 - 39 years ☐ 40 - 59 years ☐ 60 - 79 years ☐ 80 - 100 years

3. Have you ever been diagnosed with: (Tick all that apply.)

Diabetes type 1	☐ No	☐ Yes	Parkinson's Disease	☐ No	☐ Yes
Diabetes type 2	☐ No	☐ Yes	Vertigo	☐ No	☐ Yes
CVA (stroke)	☐ No	☐ Yes	Labyrinthitis	☐ No	☐ Yes
Mini Stroke (TIA)	☐ No	☐ Yes	PoTS (Postural Tachycardia Syndrome)	☐ No	☐ Yes
Multiple Sclerosis	☐ No	☐ Yes	Any condition that may affect your balance	☐ No	☐ Yes

If yes please comment:

4. Is your vision:

☐ Normal

☐ Normal when corrected by glasses

☐ Partially sighted

☐ Blind

5. Have you got an injury or a condition affecting your non-amputated side?

☐ Yes

☐ No

☐ N/A (I am a double amputee)

If yes please give details:

6. Are you on any medication that you know may have side effects that cause dizziness/loss of balance?

☐ Yes

☐ No

If yes please give details:

7. What amputation level(s) do you have? (Tick all that apply.)

☐ Partial foot or ankle disarticulation (through ankle)

☐ Transtibial (below knee)

☐ Through knee

☐ Transfemoral (above knee)

☐ Through hip or hemipelvectomy

8. How long have you been an amputee?

☐ Less than one year ☐ 1 – 5 years ☐ 6 – 10 years ☐ Over 10 years

9. What aids or support do you normally use whilst walking or for mobility? (Tick all that apply.)

☐ Prosthesis with no walking aid

☐ Prosthesis and walking stick x 1

☐ Prosthesis and walking stick x 2

☐ Prosthesis and walking frame

☐ Prosthesis and crutches x 2

☐ Prosthesis and crutch x 1

☐ Prosthesis and wheelchair (use limb for transfers or therapy only)

☐ None of the above – please state:

Please continue overleaf…

10. How would you describe your activity level? (Choose one only.)

- ☐ K0 - Cosmetic purposes only and I don’t walk
- ☐ K1 - Mostly indoors, using walking aids
- ☐ K2 - Occasional walking outdoors on mostly flat ground with or without the use of walking aids
- ☐ K3 - Walking outdoors on various terrain and at different walking speeds without the use of walking aids
- ☐ K4 - Rigorous everyday activities such as construction work or regular sporting activity

11. During the course of the last month, how often have you tripped or stumbled?

(“Trip” - catch ones foot on something leading to a stumble or fall. ”Stumble” - momentarily lose one's balance; almost fall.)

☐ None ☐ 1 - 5 times ☐ More than 5 times

12. During the course of the last month, how often have you fallen?

(“Fall” - to lose your balance and collapse to the ground.)

☐ None ☐ 1 - 5 times ☐ More than 5 times

13. When you fell were you wearing your prosthetic limb?

☐ No ☐ Yes ☐ I can’t remember ☐ N/A

14. If you fell, where were you at the time?

☐ Indoors ☐ Outdoors ☐ I have fallen both indoors and outdoors
☐ I can’t remember ☐ N/A

15. Did you injure yourself?

☐ No ☐ Yes ☐ N/A

If yes, did you seek professional medical treatment?

☐ GP ☐ Hospital ☐ Physiotherapist ☐ Other, please specify:
☐ I didn’t seek any medical treatment

16. Did you inform any of your prosthetic clinical team that you had fallen and if so, which members?

☐ No ☐ Yes ☐ N/A
☐ Prosthetist ☐ Doctor ☐ Physiotherapist ☐ Nurse ☐ Other, please specify:

Any other comments?

When completed please return to your clinician or post into the questionnaire box on reception.
