# Supplementary material for: Can microprocessor knees reduce the disparity in trips and falls risks between above and below knee prosthesis users?
Source: PLoS One. 2022 Sep 2;17(9):e0271315. doi: 10.1371/journal.pone.0271315 (PMC9439191; doi:10.1371/journal.pone.0271315)
Supplement: S2 Appendix — (PDF) [file pone.0271315.s002.pdf]

Appendix 2: Ethical approval

APPENDIX C: STHFT SERVICE REVIEW CONFIRMATION LETTER FOR PUBLICATION OR EDUCATIONAL PURPOSES

To whomever this may concern

This letter will help STHFT staff provide evidence to prospective publishers or any other relevant parties that the stated project has been classified as service review and that ethical review has been achieved.

Title of Project: EVALUATION OF THE INCIDENCES OF TRIPS, STUMBLES AND FALLS IN LIMB WEARING AMPUTEES.

Signature of project lead clinician: ALISON STENSON

Date: 30/06/2020

Evidence this project has been signed off as service review
Which incorporates ethical review having been achieved and level of risk has been assessed
Please complete the table below.

	Please tick all that apply	Please print name, sign and date
1a. Self-declared by project lead clinician as service review +/-	✓	A STENSON 30/06/2020.
1b. Directorate Governance Lead/ Directorate Executive Team	✓	RAM HARIHARAN
1c. Signed off by the Clinical Research & Innovative Office		
1d. Signed off by the Clinical Effectiveness Unit		
1e. Signed off by the Professional and Practice Development Team		

If the project materially changes from the original project description represented to the above signatory, it is the responsibility of the project lead clinician to re-present the project for further consideration. This includes self-review in the case of 1a above. Any failure to do this is the responsibility of the lead clinician.

For more information please refer to 5 Stage Governance Process for Service Review Activity, page 20 and Table 6, point 8, pages 16 – 17 on Ethical Principles Applicable to Clinical Audit & Service Review.

The Simple Rules Toolkit. - © Sheffield Teaching Hospitals NHS Foundation Trust 2018
