# Supplementary material for: Can microprocessor knees reduce the disparity in trips and falls risks between above and below knee prosthesis users?
Source: PLoS One. 2022 Sep 2;17(9):e0271315. doi: 10.1371/journal.pone.0271315 (PMC9439191; doi:10.1371/journal.pone.0271315)
Supplement: S3 Appendix — (PDF) [file pone.0271315.s003.pdf]

Ordered Logistic Regression full outputs

1. Tripping analysis

	Coef	S.E.	Wald Z	Pr(>|Z|)	Odds ratio	2.5% CI	97.5% CI
y>=2	-0.453	0.409	-1.110	0.267	0.635	0.285	1.416
y>=3	-3.001	0.452	-6.640	<0.0001	0.050	0.021	0.121
Prosthesis_type=NonMPK	0.672	0.264	2.550	0.011	1.958	1.167	3.283
Prosthesis_type=MPK	-0.302	0.407	-0.740	0.457	0.739	0.333	1.640
Gender=Female	0.029	0.293	0.100	0.921	1.029	0.580	1.826
Age=40_59_years	-0.008	0.346	-0.020	0.983	0.992	0.504	1.955
Age=60_79_years	-0.218	0.363	-0.600	0.548	0.804	0.395	1.638
Age=80_or_more_years	-0.430	0.684	-0.630	0.530	0.651	0.170	2.484
Years_of_prost_use=Less_than_5_ years	0.010	0.361	0.030	0.979	1.010	0.497	2.050
Years_of_prost_use=6_10_years	0.483	0.326	1.480	0.139	1.620	0.855	3.070
Years_of_prost_use=More_than_10_years	-0.840	0.600	-1.400	0.161	0.432	0.133	1.399
Comorbidity=Yes	-0.118	0.289	-0.410	0.683	0.889	0.505	1.566
Contralateral_limb=Yes	0.650	0.259	2.510	0.012	1.915	1.153	3.179
Medication=Yes	0.150	0.380	0.390	0.693	1.162	0.552	2.446
Walking_aid=Yes	0.687	0.289	2.380	0.017	1.987	1.129	3.499
Activity_level=K3	0.249	0.296	0.840	0.400	1.283	0.718	2.293
Activity_level=K4	0.427	0.447	0.950	0.340	1.532	0.638	3.678

2. Falling analysis

	Coef	S.E.	Wald Z	Pr(>|Z|)	Odds ratio	2.5% CI	97.5% CI
y>=2	-2.914	0.830	-3.510	0.000	0.054	0.011	0.276
y>=3	-5.471	0.917	-5.970	<0.0001	0.004	0.001	0.025
Prosthesis_type=NonMPK	1.206	0.336	3.590	0.000	3.340	1.730	6.449
Prosthesis_type=MPK	-0.385	0.689	-0.560	0.576	0.681	0.176	2.625
Gender=Female	-0.247	0.389	-0.630	0.526	0.781	0.364	1.675
Age=40_59_years	0.551	0.547	1.010	0.313	1.735	0.594	5.068
Age=60_79_years	0.072	0.575	0.120	0.901	1.074	0.348	3.317
Age=80_or_more_years	0.060	0.988	0.060	0.952	1.062	0.153	7.362
Years_of_prost_use=Less_than_5_ years	0.153	0.499	0.310	0.760	1.165	0.438	3.099
Years_of_prost_use=6_10_years	0.082	0.426	0.190	0.847	1.086	0.471	2.503
Years_of_prost_use=More_than_10_years	-0.273	0.860	-0.320	0.751	0.761	0.141	4.108
Comorbidity=Yes	0.119	0.368	0.320	0.747	1.126	0.547	2.318
Contralateral_limb=Yes	0.387	0.333	1.160	0.244	1.473	0.768	2.827
Medication=Yes	0.543	0.449	1.210	0.227	1.721	0.714	4.147
Walking_aid=Yes	0.411	0.386	1.060	0.288	1.508	0.707	3.214
Activity_level=K3	-0.059	0.386	-0.150	0.879	0.943	0.443	2.009
Activity_level=K4	0.110	0.589	0.190	0.852	1.117	0.352	3.542

3. Falling analysis of those who reported a trip

	Coef	S.E.	Wald Z	Pr(>|Z|)	Odds ratio	2.5% CI	97.5% CI
y>=2	-1.481	0.674	-2.200	0.028	0.227	0.061	0.852
y>=3	-4.200	0.776	-5.410	<0.0001	0.015	0.003	0.069
Prosthesis_type=NonMPK	1.005	0.379	2.650	0.008	2.732	1.299	5.745
Prosthesis_type=MPK	-1.067	0.858	-1.240	0.214	0.344	0.064	1.849
Gender=Female	-0.561	0.451	-1.250	0.213	0.571	0.236	1.380
Age=40_59_years	0.466	0.592	0.790	0.432	1.593	0.499	5.087
Age=60_79_years	0.212	0.615	0.340	0.731	1.236	0.370	4.127
Age=80_or_more_years	-0.340	1.062	-0.320	0.749	0.712	0.089	5.710
Years_of_prost_use=Less_than_5_ years	0.032	0.554	0.060	0.955	1.032	0.349	3.054
Years_of_prost_use=6_10_years	-0.446	0.477	-0.930	0.350	0.640	0.251	1.632
Years_of_prost_use=More_than_10_years	-0.172	1.052	-0.160	0.870	0.842	0.107	6.616
Comorbidity=Yes	0.032	0.424	0.080	0.939	1.033	0.450	2.372
Contralateral_limb=Yes	-0.068	0.380	-0.180	0.858	0.934	0.444	1.967
Medication=Yes	0.192	0.525	0.370	0.714	1.212	0.433	3.391
Walking_aid=Yes	0.495	0.436	1.130	0.257	1.640	0.698	3.856
Activity_level=K3	0.139	0.424	0.330	0.743	1.149	0.500	2.638
Activity_level=K4	0.057	0.666	0.090	0.932	1.058	0.287	3.903
